# Five-year cardiovascular outcomes in patients with chronic myeloid leukemia treated with imatinib, dasatinib, or nilotinib: A cohort study using data from a large multinational collaborative network

**DOI:** 10.3389/fcvm.2023.888366

**Published:** 2023-02-07

**Authors:** Rafael Amorim Belo Nunes, Precil Diego Miranda de Menezes Neves, Leandro Menezes Alves da Costa, Philip Bachour, Marcelo José de Carvalho Cantarelli, Gustavo Bernardes de Figueiredo Oliveira, Álvaro Avezum Jr.

**Affiliations:** ^1^Department of Cardiology, Hospital Alemão Oswaldo Cruz, São Paulo, Brazil; ^2^International Research Center, Hospital Alemão Oswaldo Cruz, São Paulo, Brazil; ^3^Department of Hematology and Bone Marrow Transplant, Hospital Alemão Oswaldo Cruz, São Paulo, Brazil

**Keywords:** cardiovascular safety, chronic myeloid leukemia, tyrosine kinase inhibitor (TKI), breakpoint cluster region-abelson (BCR-ABL), cardio-onco-hematology

## Abstract

**Background:**

Breakpoint cluster region-Abelson gene (BCR-ABL) tyrosine kinase inhibitors (TKIs) have revolutionized the treatment of patients with chronic myeloid leukemia (CML). However, concern has arisen about the cardiac safety profile of these drugs.

**Objectives:**

This study aims to compare long-term risks of adverse cardiovascular and cerebrovascular events (ACE), heart failure or left ventricular ejection fraction (LVEF) < 50%, and venous thromboembolic events (VTE) in patients with CML treated with BCR-ABL TKIs, using data from a large multinational network.

**Methods:**

Patients aged ≥ 18 years with CML treated with imatinib, dasatinib, or nilotinib without prior cardiovascular or cerebrovascular disease were included. We used propensity score matching to balance the cohorts. The 5-year cumulative incidences and hazard ratios were calculated.

**Results:**

We identified 3,722 patients with CML under treatment with imatinib (*n* = 1,906), dasatinib (*n* = 1,269), and nilotinib (*n* = 547). Patients with imatinib compared to dasatinib showed a higher hazard ratio (HR) for ACE (HR 2,13, 95% CI 1.15–3.94, *p* = 0.016). Patients with imatinib presented a lower HR than nilotinib for ACE (HR 0.50, 95% CI 0.30–0.83, *p* = 0.0074). In relation to heart failure or LVEF < 50%, patients with imatinib had a higher HR than dasatinib (HR 9.41, 95% CI 1.22–72.17, *p* = 0.03), but no significant difference was observed between imatinib and nilotinib (HR 0.48, 95% CI 0.215–1.01, *p* = 0.064).

**Conclusion:**

In this retrospective study with a large number of patients with CML, those treated with nilotinib had a higher 5-year ratio of ACE, while patients with dasatinib showed a lower ratio than patients with imatinib. The ratio of heart failure was higher in patients with imatinib than in patients with dasatinib, but not when compared to nilotinib.

## Introduction

Chronic myeloid leukemia (CML) is a myeloproliferative disorder caused by a balanced chromosomal translocation involving a fusion of the Abelson gene (ABL1) from chromosome 9q34 with the breakpoint cluster region (BCR) gene on chromosome 22q11, known as the Philadelphia chromosome, which encodes the BCR-ABL protein with protein tyrosine kinase activity (1). The advent of BCR-ABL tyrosine kinase inhibitors (TKI) was a big breakthrough in the treatment of patients with CML, improving their outcomes and quality of life. In 2001, imatinib was the first BCR-ABL TKI approved for the treatment of patients with CML (2). Posteriorly, other BCR-ABL TKIs such as dasatinib and nilotinib were also approved for this purpose.

Despite their well-known benefits for CML treatment, concerns have been raised about short- and long-term cardiac and pulmonary safety profiles of BCR-ABL TKIs. Cardiac toxicities associated with TKIs are heterogeneous and may include QT prolongation, arrhythmias, decreased left ventricular ejection fraction (LVEF), congestive heart failure, acute coronary syndrome, arterial thrombosis, pulmonary, and systemic hypertension (3–10). TKI-induced cardiotoxicity mechanisms are miscellaneous and may be drug-specific, even to same-class drugs. Proposed mechanisms may include disruption of mitochondrial function within the cardiomyocyte, off-target inhibition of other kinases, disturbing cardiomyocyte cellular oxidative phosphorylation, and caspase-mediated mitochondrial apoptosis (11).

Despite data from clinical trials and cohort studies, there is still a need for more robust information about the long-term cardiac safety profile of BCR-ABL TKIs, especially real-world data. Trials evaluating BCR-ABL TKIs in patients with CML have enrolled participants with a history of cardiovascular disease but, when cardiac endpoints were reported, these patients were not analyzed as a separate group (12). Also, the definition of cardiovascular outcomes in TKI studies was heterogeneous and generally, they were not the same as those of cardiologic trials as well as they were not specifically designed to determine cardiac safety.

Based on the previous literature data, we hypothesized that cardiovascular outcomes may differ according to the treatment with specific BCR-ABL TKIs. In this study, we aimed to evaluate the 5-year incidence and compare the ratios of significant cardiovascular outcomes in patients with CML without a past history of heart or cerebrovascular diseases treated with Bcr-ABL TKIs imatinib, dasatinib, or nilotinib, using data from a large multinational network based on electronic medical records.

## Materials and methods

### Data source

We used global-based data from the network TriNetX (TriNetX, Inc., Cambridge, MA, United States), a multinational collaborative clinical research platform, that collects real-time medical records, including demographics, diagnoses, procedures, medications, laboratory values, and vital statuses. This network included 70 healthcare organizations at the time of analysis, including data from around 69.8 million patients. The TriNetX platform uses aggregated counts and statistical summaries of de-identified information so that no protected health information or personal data are made available to users of the platform. Data were extracted and analyzed from the Global Collaborative Network on the TriNetX platform between 27 August and 30 August 2021.

### Study population

We queried the databank to select patients of both sexes and with age ≥ 18 years with CML, BCR-ABL positive, based on International Classification of Diseases, Tenth Revision (ICD-10) diagnosis codes (ICD-10 code C92.1) during the past 10 years before the analysis. The patients needed to be under treatment with a BCR-ABL TKI (imatinib, dasatinib, or nilotinib), and they must not be prescribed another TKI anytime. The index date was determined by the earliest date of identification of the use of a BCR-ABL TKI. Patients with past ischemic heart disease, other forms of heart disease (including patients with LVEF < 50% identified in the TriNetX databank), cerebrovascular disease, and pulmonary hypertension (ICD-10 codes I20-I25, I27, I30-I52, and I60-I69) before the index date were excluded from the analysis.

### Study design

In this population-based retrospective cohort study, we aimed to analyze the 5-year incidence of cardiovascular outcomes [adverse cardiovascular and cerebrovascular events (ACE), heart failure or LVEF < 50%, and venous thromboembolic events (VTE)] and their comparative hazard ratios (HR) in patients with CML BCR-ABL positive under treatment with imatinib, dasatinib, or nilotinib. The time window for the outcome was the treatment starting with a BCR-ABL TKI up to 5 years after. To avoid interactions in cardiovascular outcomes from patients with CML that were changed from one TKI to another, patients with switch therapy were excluded from the analysis.

### Outcomes

The three analyzed cardiovascular outcomes are as follows:

**Adverse cardiovascular and cerebrovascular events (ACE):** the composite of ischemic heart disease (ICD-10 codes I20–I25) or cerebrovascular disease (ICD-10 codes I60–69) or myocardial revascularization (coronary angioplasty code or coronary artery bypass graft surgery, ICD-10 codes Z95.1, Z95.5, and Z98.61).
**Heart failure or left ventricular ejection fraction (LVEF) < 50%:** ICD-10 code I50 or TriNetX code 2003. LVEF < 50% was chosen as a surrogate for ventricular dysfunction.
**Venous thromboembolism (VTE):** ICD-10 I82 or I26.

### Statistical analysis

The baseline characteristics for each group were compared with the chi-square test for categorical variables and the Student *t*-test for continuous variables. Propensity score matching was used to balance cohorts with baseline characteristics. In relation to outcome comparisons, we used the imatinib group as the reference, comparing it with the two other groups (dasatinib and nilotinib). Kaplan–Meier analysis was performed to estimate the probability of outcomes after the index date from 1 day up to 5 years. Comparisons between cohorts were made using a log-rank test. We calculate the HRs and their associated 95% confidence intervals (CI), together with the test for proportionality based on the scaled Schoenfeld residual, using R’s Survival package v3.2-3.

Statistical analyses were done within TriNetX (13). Statistical significance was set at a two-sided *p*-value of < 0.05.

### Propensity score matching and covariates

The propensity score matching was calculated using logistic regression implemented by the function logistic regression of the scikit-learn package in Python version 3.7. “Greedy nearest neighbor matching” was used with a caliper of 0.1 pooled standard deviations (13). 1:1 matching was adjusted for covariates that could be confounders for the predefined cardiovascular outcomes as follows: demographic variables such as age, sex, and race (defined as white, black or African American, Asian, or unknown); health conditions related to cardiovascular risk and recorded identified from ICD-10-CM codes in electronic medical records: overweight or obesity, hypertension, chronic kidney disease, dyslipidemia, diabetes mellitus, and nicotine dependence; and use of cardiovascular and antimetabolite medications: hydroxyurea, diuretics, ACE inhibitors or angiotensin II receptor blockers, beta-blockers, lipid-lowering drugs, and antiarrhythmics, before starting BCR-ABL TKIs.

### Ethics

TriNetX-derived studies with de-identified information were approved by the Institutional Review Board of Hospital Alemão Oswaldo Cruz.

## Results

### Characteristics of the study population

Using the electronic medical records from the platform TriNetX, we identified 24,921 patients with CML BCR/ABL positive (ICD-10 C92.1). From this cohort, we selected, using inclusion and exclusion criteria, 3,722 patients with CML and without past heart disease treated with imatinib (*n* = 1,906), dasatinib (*n* = 1,269), and nilotinib (*n* = 547). The exposure time for each analyzed TKI was as follows: imatinib (median 1,198 days, range 1–1,826 days), dasatinib (median 647 days, range 1–1,826 days), and nilotinib (median 790 days, range 1–1,826 days).

Compared to imatinib, patients with dasatinib were younger during the start of treatment with a TKI (age 55 vs. 47.7 years old, *p* < 0.0001), had a lower rate of hypertension (21 vs. 16%, *p* < 0.0001), and had diabetes mellitus (10 vs. 7%, *p* = 0.002). The patients with dasatinib had a higher rate of previous treatment with hydroxyurea (*p* < 0.0001) and antiarrhythmics (*p* < 0.0001) than patients in the imatinib group.

Compared to the imatinib group, patients from the nilotinib group were younger (55 vs. 53 years old, *p* = 0.008), had a higher proportion of female patients (46 vs. 52%, *p* = 0.007), and had a higher proportion of black or African American patients (11 vs. 15%, *p* = 0.02). Patients with nilotinib had a lower rate of use of antiarrhythmics than patients with imatinib. The baseline characteristics before propensity score matching of the three study groups are detailed in Table 1.

**TABLE 1:** Baseline characteristics of the cohort for the imatinib, dasatinib, and nilotinib groups.

	Imatinib	Dasatinib	*P*-value*	Nilotinib	*P*-value**
Cohort size, *n*	1906	1269		547	
**Demographics**					
Mean age (SD), years	55 (16)	47.7 (15)	<0.0001	53 (15)	0.0086
**Sex**					
Male, *n* (%)	1029 (54)	700 (55)	NS	261 (48)	0.0092
Female, *n* (%)	877 (46)	569 (45)	NS	285 (52)	0.0073
**Race**					
White, *n* (%)	1323 (69)	820 (65)	0.0281	362 (66)	NS
Black or African American, *n* (%)	215 (11)	185 (15)	0.0026	81 (15)	0.0230
Asian, *n* (%)	35 (2)	30 (2)	NS	10 (2)	NS
Unknown, *n* (%)	323 (17)	223 (18)	NS	97 (18)	NS
**Comorbidities**					
Overweight or obesity, *n* (%)	133 (7)	75 (6)	NS	30 (5)	NS
Nicotine dependence, *n* (%)	113 (6)	79 (6)	NS	29 (5)	NS
Disorders of lipid metabolism, *n* (%)	228 (12)	117 (9)	NS	61 (11)	NS
Diabetes mellitus, *n* (%)	187 (10)	85 (7)	0.0002	57 (10)	NS
Hypertension, *n* (%)	401 (21)	202 (16)	<0.0001	96 (18)	NS
Chronic kidney disease, *n* (%)	79 (4)	28 (2)	<0.0001	13 (2)	NS
**Medications**					
Hydroxyurea, *n* (%)	252 (13)	328 (26)	<0.0001	66 (12)	NS
Antilipemic agentes, *n* (%)	311 (16)	164 (13)	0.002	92 (17)	NS
Beta blockers, *n* (%)	269 (14)	124 (10)	<0.0001	87 (16)	NS
ACE inhibitors, *n* (%)	200 (10)	174 (14)	0.0002	57 (10)	NS
Angiotensin II inhibitors, *n* (%)	127 (7)	62 (5)	0.003	34 (6)	NS
Antiarrhthymics	362 (19)	317 (25)	0.0002	56 (10)	0.0001
Diuretics, *n* (%)	323 (17)	165 (13)	<0.0001	70 (13)	0.018

SD, standard deviation; ACE, angiotensin-converting enzyme; NS, non-significant. *imatinib vs. dasatinib; **imatinib vs. nilotinib.

### Outcome incidences during 5-year follow-up before the propensity score matching

The number and cumulative incidence of patients with ACE in the Imatinib group were 99 (5.23%), in the dasatinib group were 15 (1.19%), and in the nilotinib group were 44 (8.1%). For the composite outcome heart failure or LVEF < 50%, the number and cumulative incidence in the imatinib group were 35 (1.83%), in the dasatinib group were 10 (0.78%), and in the nilotinib group were 17 (3.1%). The composite outcome VTE or pulmonary embolism occurred in 45 (2.4%) patients in the imatinib group, 25 (2%) patients in the dasatinib group, and 10 (1.8%) patients in the nilotinib group.

### 5-year outcomes in imatinib, dasatinib, and nilotinib groups after propensity score matching

**FIGURE 1:**
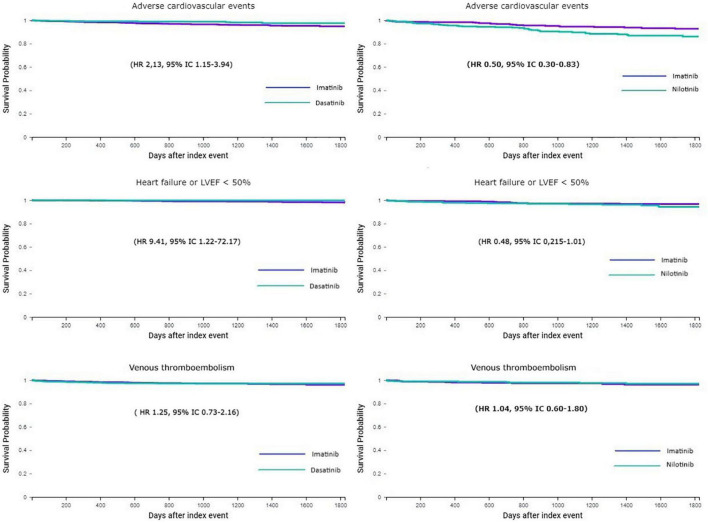
5-year Kaplan–Meier curves for adverse cardiovascular or cerebrovascular events, heart failure or LVEF < 50%, and VTE or pulmonary embolism in patients treated with imatinib vs. dasatinib (**left** column) and imatinib vs. nilotinib (**right** column). LVEF, left ventricular ejection fraction; HR, hazard ratio; CI, confidence interval; VTE, venous thromboembolism.

After the propensity score matching, the imatinib group (*n* = 1,153) compared to the dasatinib group (*n* = 1,153) showed a higher HR for ACE (HR 2.13, 95% CI 1.15–3.94, *p* = 0.016). When compared with the nilotinib group (*n* = 533), the matched imatinib group (*n* = 533) presented a lower HR for ACE (HR 0.50, 95% CI 0.30–0.83, *p* = 0.0074). In relation to heart failure or LVEF < 50%, patients with imatinib had a higher ratio than patients with dasatinib (HR 9.41, 95% CI 1.22–72.17, *p* = 0.03), but no significant difference was observed between imatinib and nilotinib groups (HR 0.48, 95% CI 0.215–1.01, *p* = 0.064). There were no significant differences between the three groups in relation to VTE or pulmonary embolism. Five-year Kaplan–Meier curves for ACE, heart failure, and venous thromboembolism between dasatinib vs. imatinib and nilotinib vs. imatinib are depicted in Figure 1.

## Discussion

We used a large electronic medical record network to create propensity score-matched cohorts of patients with CML without a past history of heart or cerebrovascular diseases according to the treatment with three commonly used BCR-ABL TKIs for comparing ratios of cardiovascular outcomes (ACE, heart failure or LVEF < 50%, and VTE or pulmonary embolism) during a 5-year period. As patients of the imatinib group were older than patients of the other groups and had more cardiovascular risk factors than patients of the dasatinib group, we opted for matching the cohorts as a reasonable approach to compare the groups with similar baseline characteristics for reducing bias. Using this approach, we found that when compared with patients from the imatinib group, patients with dasatinib had a significantly lower ratio of ACE, while patients from the nilotinib group had a significantly higher rate of ACE.

Cardiovascular events, including ischemic heart disease, cerebrovascular disease, and peripheral artery disease, are major concerns in patients with CML, particularly in those under treatment with second- and third-generation Bcr-ABL TKIs nilotinib and ponatinib, respectively (14). In this population, cardiovascular disease may be responsible for up to 16.5 and 5% of potential years of life lost in men and women, respectively (15). In the 3-year follow-up of the ENESTnd trial, which included a total of 846 patients with newly diagnosed Ph + CML-CP, the incidence of ischemic heart disease was higher with nilotinib than with imatinib: nine patients (3.2%) in the nilotinib 300 mg twice-daily arm, 11 patients (4.0%) in the nilotinib 400 mg twice-daily arm, and three patients (1.1%) in imatinib arm (9). These results were more evident with a 5-year update of the ENESTnd trial, in which 28 of 279 (10%) patients treated with nilotinib at 300 mg twice per day, 44 of 277 patients (15.9%) treated with nilotinib at 400 mg twice per day, and 7 (2.5%) of 280 patients treated with imatinib 400 once per day had ischemic events (16). Interestingly, a retrospective study using data from 1,390 patients with CML from IRIS, TOPS, and ENESTnd trials showed a lower risk of peripheral arterial disease in patients treated with imatinib vs. nilotinib or patients with CML treated without TKIs, suggesting a possible protective role of imatinib in atherosclerotic vascular diseases (10). The supposed mechanisms related to nilotinib cardiovascular toxicities are complex and may involve different pathways. Nilotinib may lead to overexpression of cell adhesion proteins on human endothelium, including ICAM1, VCAM1, and E-selectin, which may enroll inflammatory cells and platelets and increase the risk of cardiovascular events (14). Nilotinib also represses endothelial cell proliferation and migration and may inhibit other kinases related to angiogenesis (17). In our study, in line with previous data, we have also observed a higher risk of cardiovascular disease, including cerebrovascular and ischemic heart disease or myocardial revascularization, in patients with nilotinib when compared with imatinib, even excluding patients with a past history of heart and cerebrovascular disease and adjusting the analysis for baseline cardiovascular risk factors.

The risk of cardiovascular ischemic events induced by dasatinib is not well-established. Despite the higher incidence of cardiovascular events in patients treated with dasatinib in relation to imatinib (4 vs. 2%) at the 5-year follow-up of the DASISION trial (8), a *post hoc* analysis that included patients from the DASISION trial showed that the cardiovascular events occurred mainly in patients with a history of cardiovascular disease (18). In a retrospective study that analyzed data from 105 patients with CML in Polish tertiary health centers, patients treated with dasatinib had lower rates of vascular events (4%) than patients with nilotinib (11%) (19). We showed in our analysis a lower HR of cardiovascular and cerebrovascular events in patients with dasatinib compared to imatinib, differing from other analyses by being a real-world cohort and excluding patients with overt cardiovascular and cerebrovascular diseases before the start of a TKI. It is also essential to keep in mind the TKIs used for CML treatment differ in their potency and activity against BCR-ABL1 and other kinases, which also exert relevant functions on the cardiovascular system. This can in part explains the observed discrepancy in the cardiovascular risk between the different TKIs.

A warning signal for the risk of heart failure in patients with CML treated with BCR-ABL TKIs was suggested in 2006 by Kerkelä and colleagues when reporting a case series of 10 patients treated with imatinib that had developed heart failure with reduced LVEF. Myocardial biopsies in two patients and three imatinib-treated mice showed mitochondrial abnormalities and accumulation of membrane whorls in both vacuoles and sarcoplasmic reticulum, suggesting toxic myopathy possibly associated with ABL inhibition (20). However, a further follow-up study of patients treated with imatinib did not demonstrate a higher risk of heart failure or cardiomyopathy (21). In our cohort, the unmatched incidences of the composite outcome heart failure or LVEF < 50% were higher in the imatinib group (1.83%) and the nilotinib group (3.1%) than in the dasatinib group (0.78%). After matching for baseline risk factors, patients treated with imatinib showed a greater ratio for heart failure or a reduced LVEF than patients with dasatinib but not in relation to patients with nilotinib, suggesting that in a long term, dasatinib may exert less toxicity on ventricular function than imatinib or nilotinib. However, we should take into account that heart failure incidence was low in the three treatment groups.

In our study, patients with CML who were switched from one TKI to another were excluded from the analysis. One might question that patients on dasatinib who stopped medication for pleural effusion, which is a common adverse event with this medication, might have a higher incidence of heart failure and lead to a selection bias. However, the physiopathology of pleural effusion in patients treated with dasatinib is not related to heart failure, given that dasatinib-induced pleural effusions are generally lymphocyte-predominant exudates (22). In addition, data indicate dasatinib-induced pleural effusion could be related to strong inhibition of the platelet-derived growth factor receptor β, leading to a decreased interstitial fluid pressure and higher vascular permeability (23).

Venous thromboembolic events have not been described as a significant adverse effect of BCR-ABL TKIs. An exception was a phase 2 study that included patients with CML for ponatinib treatment, which showed moderate rates of VTE, mostly deep vein thrombosis and pulmonary embolism, that occurred in 5% of patients (24). In our study, the overall incidence of VTE or pulmonary embolism was low in the three TKI groups, and we have not observed a difference in risk between them.

The strengths of this study are the sample size, considering the low incidence of CML in many healthcare organizations, the capacity of adjusting the outcomes for baseline cardiovascular characteristics, and the real-world essence of the data. We must recognize several limitations of this study. First, it involved analyses of a retrospective observational cohort, which led to baseline differences among the treatment arms, such as older individuals with more cardiovascular risks in the imatinib group. This fact led us to use propensity score matching, which may have some problems like trying to mimic a randomized experiment, without the same precision and control against confounding. Also, propensity score matching may create a “propensity score paradox,” in which unit pruning causes increased imbalance after a point (25).

Due to its observational retrospective nature, the study may be inherently subject to bias. Therefore, we should carefully avoid making cause–effect relationships and, instead, consider the results as a hypothesis generator. Second, despite the matching, we cannot exclude the influence of residual confounding, which was not captured, such as TKIs and cardiovascular medications dosage, administration timing since the beginning of the follow-up, and lifestyle habits such as physical activity and diet. Particularly TKI dosage may be an important factor for cardiotoxicity as showed in the ENESTnd trial, in which a higher dosage of nilotinib was associated with a higher incidence of arterial events in relation to a lower dosage or with imatinib (15). We were also unable to analyze the treatment discontinuation rates for the three BCR-ABL TKI groups, which could influence the timing of exposition to the TKI and their cardiovascular effect. Third, the outcomes and baseline characteristics were based on 10 ICD codes, which are not accurate when compared with adjudicated outcomes in randomized clinical trials.

In conclusion, we found in a large sample of patients with CML treated with BCR-ABL TKIs that when compared with imatinib treatment, patients treated with nilotinib had a higher ratio of ACE, while patients treated with dasatinib showed a lower ratio of cardiovascular and cerebrovascular events. The ratio of heart failure was greater in patients with imatinib when compared to dasatinib but not compared to nilotinib. These results raise the hypothesis that, when comparing three commonly used BCR-ABL TKIs, nilotinib presents a higher probability of cardiovascular toxicity and dasatinib presents a better cardiovascular safety profile. These findings may be particularly relevant in patients with CML and underlying cardiovascular risk factors, in which a BCR-ABL TKI is being considered for treatment.

## Data availability statement

The original contributions presented in this study are included in the article/supplementary material, further inquiries can be directed to the corresponding author.

## Ethics statement

The studies involving human participants were reviewed and approved by Comissão de Ética em Pesquisa do Hospital Alemão Oswaldo Cruz. Written informed consent for participation was not required for this study in accordance with the national legislation and the institutional requirements.

## Author contributions

All authors listed have made a substantial, direct, and intellectual contribution to the work, and approved it for publication.
